# Association of Developmental Language Disorder With Comorbid Developmental Conditions Using Algorithmic Phenotyping

**DOI:** 10.1001/jamanetworkopen.2022.48060

**Published:** 2022-12-29

**Authors:** Rachana Nitin, Douglas M. Shaw, Daniel B. Rocha, Courtney E. Walters, Christopher F. Chabris, Stephen M. Camarata, Reyna L. Gordon, Jennifer E. Below

**Affiliations:** 1 Vanderbilt Brain Institute, Vanderbilt University, Nashville, Tennessee; 2 Department of Otolaryngology–Head & Neck Surgery, Vanderbilt University Medical Center, Nashville, Tennessee; 3 Vanderbilt Genetics Institute, Vanderbilt University Medical Center, Nashville, Tennessee; 4 Phenomic Analytics and Clinical Data Core, Geisinger, Danville, Pennsylvania; 5 NewYork-Presbyterian Hospital, New York; 6 Vanderbilt University Neuroscience Program, Vanderbilt University, Nashville, Tennessee; 7 Loma Linda School of Medicine, Loma Linda University, Loma Linda, California; 8 Geisinger Health System, Lewisburg, Pennsylvania; 9 Department of Hearing and Speech Sciences, Vanderbilt University Medical Center, Nashville, Tennessee; 10 Department of Psychology, Vanderbilt University, Nashville, Tennessee; 11 Vanderbilt Kennedy Center, Vanderbilt University Medical Center, Nashville, Tennessee; 12 Department of Medicine, Vanderbilt University Medical Center, Nashville, Tennessee

## Abstract

**Importance:**

Developmental language disorder (DLD) is a common (with up to 7% prevalence) yet underdiagnosed childhood disorder whose underlying biological profile and comorbidities are not fully understood, especially at the population level.

**Objective:**

To identify clinically relevant conditions that co-occur with DLD at the population level.

**Design, Setting, and Participants:**

This case-control study used an electronic health record (EHR)–based population-level approach to compare the prevalence of comorbid health phenotypes between DLD cases and matched controls. These cases were identified using the Automated Phenotyping Tool for Identifying Developmental Language Disorder algorithm of the Vanderbilt University Medical Center EHR, and a phenome enrichment analysis was used to identify comorbidities. An independent sample was selected from the Geisinger Health System EHR to test the replication of the phenome enrichment using the same phenotyping and analysis pipeline. Data from the Vanderbilt EHR were accessed between March 2019 and October 2020, while data from the Geisinger EHR were accessed between January and March 2022.

**Main Outcomes and Measures:**

Common and rare comorbidities of DLD at the population level were identified using EHRs and a phecode-based enrichment analysis.

**Results:**

Comorbidity analysis was conducted for 5273 DLD cases (mean [SD] age, 16.8 [7.2] years; 3748 males [71.1%]) and 26 353 matched controls (mean [SD] age, 14.6 [5.5] years; 18 729 males [71.1%]). Relevant phenotypes associated with DLD were found, including learning disorder, delayed milestones, disorders of the acoustic nerve, conduct disorders, attention-deficit/hyperactivity disorder, lack of coordination, and other motor deficits. Several other health phenotypes not previously associated with DLD were identified, such as dermatitis, conjunctivitis, and weight and nutrition, representing a new window into the clinical complexity of DLD.

**Conclusions and Relevance:**

This study found both rare and common comorbidities of DLD. Comorbidity profiles may be leveraged to identify risk of additional health challenges, beyond language impairment, among children with DLD.

## Key Points

**Question:**

Can phecode information from electronic health records (EHRs) be leveraged to identify comorbidities of developmental language disorder (DLD)?

**Findings:**

In this case-control study of 5273 DLD cases and 26 353 matched controls selected from a large EHR, both common and rare comorbidities of DLD were identified.

**Meaning:**

The findings of this study suggest that EHRs can be successfully mined to identify both rare and common traits in the medical phenome associated with DLD at the population level.

## Teaser

This case-control study identifies relevant phenotypes associated with developmental language disorder in a pediatric patient population.

## Introduction

Developmental language disorder (DLD) is a commonly occurring (with up to 7% prevalence) pediatric communication disorder characterized by difficulty in effectively learning and using spoken language, especially vocabulary grammar, with male children being at a slightly higher risk than female children (1.33:1 ratio of males to females diagnosed with DLD).^1,2,3^ Although a prevalent disorder, DLD is often underdiagnosed, with almost three-fourths of children with language-related impediments not receiving proper diagnosis or treatment.^1,4,5,6^

Developmental language disorder is a well characterized disorder that is marked by impairments in receptive and expressive language that manifest as struggles with following multistep instructions, difficulty holding coherent conversations and expressing themselves, and impairments in reading and writing skills.^7^ Language development issues in children with DLD include late-onset speaking and communication; difficulty with syntax use, vocabulary, word finding, and semantics; immature morphological formation; impaired lexicon; and slower verbal learning and memory in receptive and/or expressive language domains. Identification of delay in typical language development can occur as early as 18 months of age, during routine developmental testing administered in the physician’s office. A DLD diagnosis requires special testing for language and development, which is usually performed by clinical experts, such as speech-language pathologists, developmental specialists, pediatricians, and clinical psychologists.

Developmental language disorder occurs independent of external trauma, neurodegeneration, abuse, or brain damage. In addition, children with DLD are phenotypically distinguishable from individuals whose language capabilities are affected by alternative phenotypes, including hearing loss, autism spectrum disorder (ASD), cerebral palsy, global intellectual disability, and other known neurodevelopmental disorders.^8,9,10,11^ Even though the term *DLD* has been in use for only about a decade,^5^ substantial idiopathic impairments in language development without apparent intellectual difficulties have been recognized since the early 1800s.^12^ Although DLD is 7 times more likely to occur than ASD and almost 40 times more prevalent than pediatric hearing impairments,^4^ DLD has been understudied.^4^

As with ASD and attention-deficit/hyperactivity disorder (ADHD), DLD is often accompanied by several other comorbidities. Previous evidence has indicated that the neuropsychiatric profiles of children and adults with DLD are fairly complex.^13^ Attention-deficit/hyperactivity disorder is a commonly co-occurring condition that affects children with DLD and has further implications for educational trajectories.^14,15,16,17,18^ Some studies have also found that children with DLD face issues with mathematical and reading tasks, indicating an overlap with developmental dyslexia and dyscalculia.^19,20,21,22^ These patterns of comorbidities have been replicated in studies wherein adults who were diagnosed with severe DLD in childhood showed continued reduction in literacy and cognitive skills,^23^ decreased employability^24^; and overall issues associated with low self-esteem and self-reported detriments toward emotional health, especially depression and anxiety.^25^ Individuals who were diagnosed with DLD in childhood will often show continued impairments in their cognitive profiles, including higher rates of schizotypal features, well into adulthood.^26^ The outcomes of such comorbidities include low verbal IQ, persistent literacy delays, higher rates of unemployment, and difficulties with social adaptability.^26,27,28,29^

Developmental language disorder does not have a single unique diagnostic billing code; thus, to label DLD cases, we leveraged an existing electronic health record (EHR)–based tool with high positive predictive value (90%-95%) for DLD called the Automated Phenotyping Tool for Identifying Developmental Language Disorder (APT-DLD).^30^ We then performed an enrichment analysis to identify comorbidities across the medical phenome. The aim of this study was to identify these clinically relevant comorbid conditions that co-occur with DLD using data-rich EHRs to better understand the epidemiological characteristics of DLD at a population level and potentially raise awareness of the DLD phenotype in the clinical community.^5^

## Methods

This case-control study leveraged deidentified archival patient data from an EHR system to identify clinically relevant comorbid conditions of DLD using an enrichment analysis approach. The Vanderbilt University Medical Center Institutional Review Board deemed this study exempt from review and waived the informed consent requirement because of its use of archival, deidentified data. We followed the Strengthening the Reporting of Observational Studies in Epidemiology (STROBE) reporting guideline.

### Identifying DLD Cases From EHRs

The DLD cases for both the primary enrichment analysis and the replication analysis were identified using the APT-DLD phenotyping algorithm (eMethods in Supplement 1). Generally, case identification in EHRs relies on *International Classification of Diseases* (*ICD*) codes, which denote patient status. However, there is no singular *ICD* code assigned to DLD.^31^ The APT-DLD algorithm was developed to identify cases of DLD in EHRs.^30^

### Study Population

**Figure 1.**
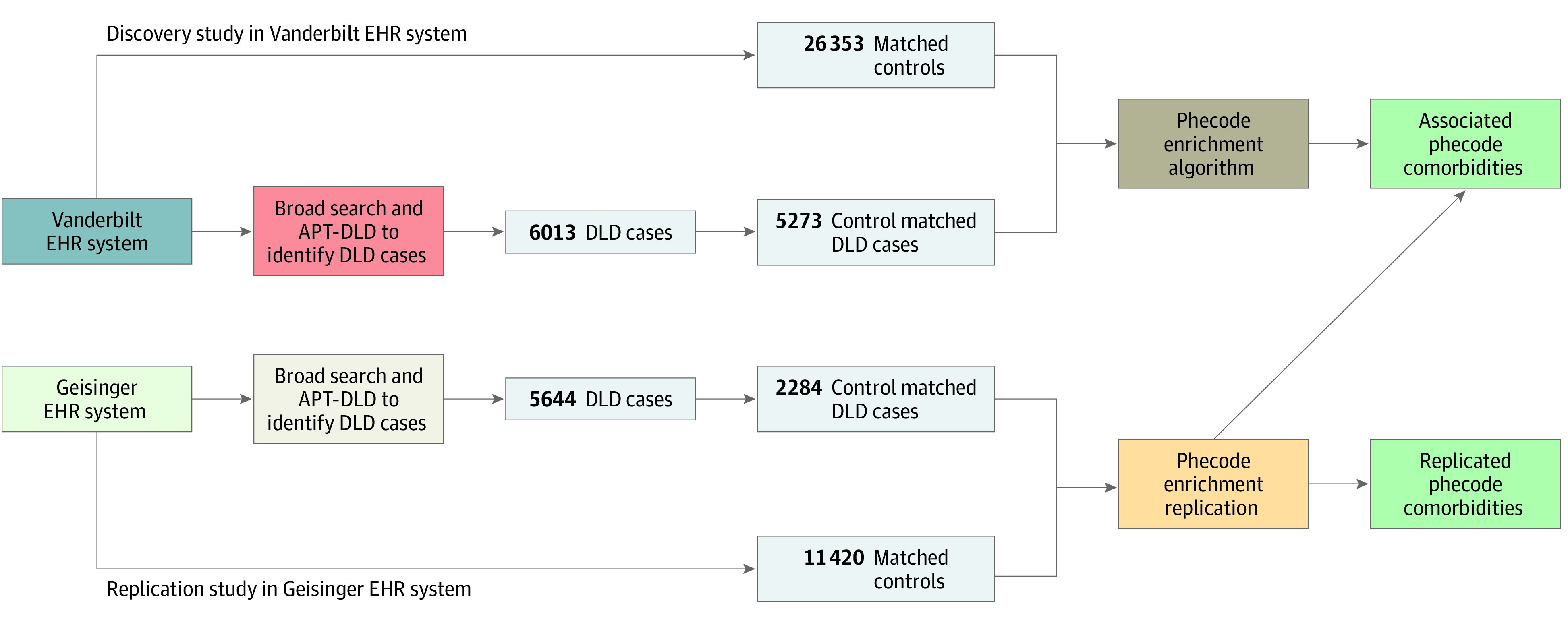
Study Design The primary analyses used the Vanderbilt University Medical Center’s Synthetic Derivative electronic health record (EHR) system, and the replication analysis was performed using the Geisinger EHR system. Records of pediatric patients with developmental language disorder (DLD) were identified using the Automated Phenotyping Tool for Identifying Developmental Language Disorder (APT-DLD), after which phecode enrichment analysis was performed to identify DLD-associated comorbidities.

The DLD case set was previously identified by applying the APT-DLD algorithm to the 3.5 million clinical records in Vanderbilt University Medical Center’s EHR system called Synthetic Derivative, as part of the APT-DLD development efforts; the details were reported in Walters et al.^30^ The APT-DLD algorithm identified 6013 pediatric records as DLD cases with available *ICD* codes, dates of the *ICD* codes, and relevant demographic information (Table 1). This cohort of DLD cases was used as the input for the large-scale comorbidity analysis performed in the present study. Data from Synthetic Derivative were accessed between March 2019 and October 2020 (Figure 1).

**Table 1.** Demographic Characteristics of the Sample Identified From the Synthetic Derivative

Variable	No. (%)	
	Controls	DLD cases
No. of records	26 353	5273
Sex^a^		
Male	18 729 (71.1)	3748 (71.1)
Female	7615 (28.9)	1528 (29.0)
Race and ethnicity		
African American	5109 (19.4)	1018 (19.3)
Asian	477 (1.8)	95 (1.8)
White	11 706 (44.4)	2320 (44.0)
Unknown	9061 (34.4)	1840 (34.9)
Mean (SD) age of records, y	16.8 (7.2)	14.6 (5.5)
Mean (SD) No. of visits	8.4 (14.21)	8.1 (15.1)

Abbreviation: DLD, developmental language disorder.

^a^ Nine controls and 2 cases were classified as unknown sex.

We identified the control set from a pool of 700 000 EHRs with available demographic and phecode records (phecode records were similarly mapped from only *International Classification of Diseases, Ninth Revision* [*ICD-9*] codes) randomly selected from Synthetic Derivative. These controls were selected to match the case set based on ethnicity and race, age (within 5 years of the matched case), and number of clinical visits (within 20 visits of the matched case) to control for quantity of health care usage. Ethnicity and race of the cases and controls were collected from the information provided within each EHR. Clinical visits were estimated by tallying the number of unique days that a phecode was acquired for each patient, with phecodes being noted exclusively from their *ICD-9* codes. The DLD cases generally had an inflated visit count compared with the controls, often because of treatment for their language disorder. To account for this, we counted any clinical visit that included only the diagnoses related to DLD and only if it was the patient’s first visit. Each subsequent visit was required to include at least 1 phecode in addition to a 315.2 phecode (speech and language disorder) within the visit count.

Up to 5 controls were selected for each case. Cases unable to be matched to a single control based on the criteria were removed from this study. After control selection, 5273 cases and 26 353 controls were selected for the study. Of this case set, 5267 cases were matched with 5 controls, 3 cases were matched with 4 controls, 1 case was matched with 3 controls, 1 case was matched with 2 controls, and 1 case was matched with 1 control.

### Statistical Analysis

#### Comorbidity Analysis via Phecode Enrichment

Given that there are tens of thousands of *ICD* codes, many of which are highly specific or overlapping, these *ICD* billing codes have been generalized and clustered into phecodes based on clinical similarity and common etiology. Phecodes represent broad categories of similar and co-occurring *ICD* billing codes based on the Phecode Map 1.2, as outlined in the phenomewide association study catalog and by Denny et al.^32^ Phecode-based analyses have been used to detect comorbidities in ASD,^33^ stuttering,^34^ and multiple sclerosis.^35^ Phecodes for the DLD case set were mapped exclusively from their *ICD-9* codes (excluding *International Statistical Classification of Diseases and Related Health Problems, Tenth Revision* [*ICD-10*] codes) to 806 corresponding phecodes, which represent broad phenotype categories that capture commonalities in the *ICD* codes to group together otherwise extremely specific diseases or disorders.^32^

**Figure 2.**
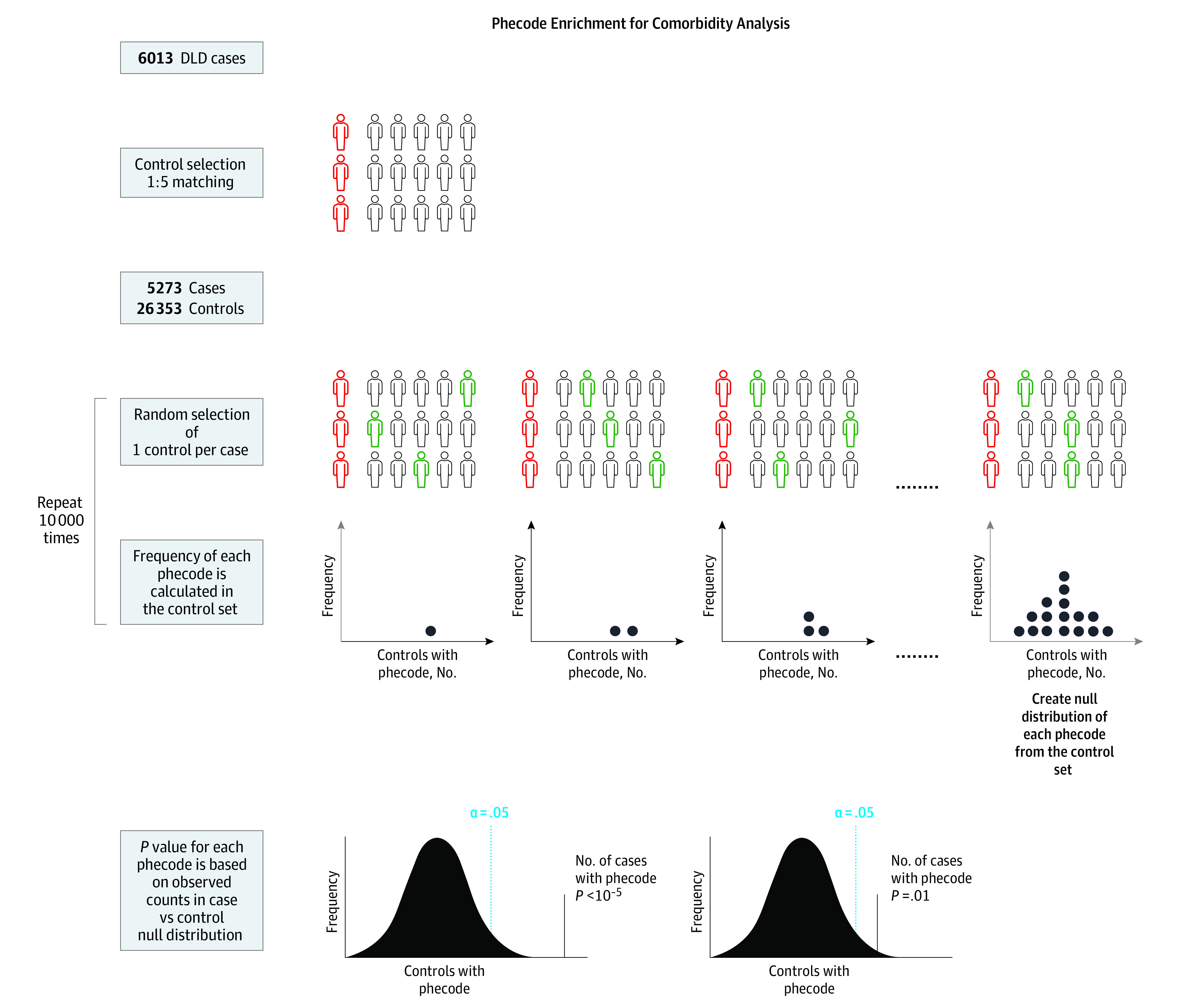
Overview of the Phecode Enrichment Algorithm Used for Comorbidity Analysis Matched controls were selected for the 6013 cases with developmental language disorder (DLD), leaving 5273 cases and 26 353 controls. *International Classification of Diseases* codes were mapped to their respective phecodes. One control per case was selected at random, and a null distribution of each phecode in the case set was created from the control group; this step was repeated 10 000 times. A *P* value was assigned to each phecode based on the observed counts of that phecode in the cases vs the counts calculated over the 10 000 permutations, to determine whether the phecode was statistically enriched in the cases vs controls.

To identify conditions that were comorbid with DLD identified from the EHRs, we performed a phecode enrichment analysis^34^ using R, version 3.5.1 (R Foundation for Statistical Computing) to calculate an empirical *P* value by comparing the overall frequency for each phecode within the case set with a null phecode frequency distribution calculated through multiple permutations of a randomized control set selected from the matched control pool (Figure 2). The R script for the phecode enrichment is provided in the eMethods in Supplement 1. For each permutation, a set of controls matching the case sample size was selected at random. To ensure that the overall control demographic structure remained similar to that of the case set, we selected only 1 control from each case’s matched control set. Phecodes for each selected control were then counted to create tallies for all defined phecode clusters. This method was then repeated for 1 × 10^5^ permutations, and a null distribution for each phecode within the control set was created.

A *P* value for each phecode was calculated via an unpaired, 2-tailed, 1-sample *t* test to determine if the prevalence of each phecode in DLD cases was significantly greater than the permutation-derived empirical null distribution. To account for multiple testing, we applied Bonferroni correction for 806 phecodes (α was set at *P* = 6.2 × 10^−5^). A phecode was significantly enriched if *P* < 6.2 × 10^−5^. The *ICD* codes used for case assignment by the APT-DLD algorithm were excluded from all phecode enrichment analyses. Data analyses were conducted between December 2019 and April 2022.

#### Replication Analysis

We sought to independently replicate the phenome enrichment in a second independent cohort at Geisinger Health System. Geisinger’s research data warehouse contains deidentified EHR data for over 2.2 million patients. We deployed the APT-DLD algorithm in the Geisinger EHR system and identified 12 591 records that met the broad search criteria, from which the APT-DLD algorithm classified 3638 records as DLD cases. To select controls for this set of DLD cases, we used all records that did not have a 315.2 phecode (encompassing several language-related *ICD* codes) as the primary pool of records. From this pool, using identical control-matching criteria as for the DLD cohort from Synthetic Derivative, we selected 5 controls per case. The final DLD case-control set for the replication analysis consisted of 3631 DLD cases and 18 155 matched controls. Data from the Geisinger EHR were accessed between January and March 2022.

Using the list of phecodes that were associated or enriched with Synthetic Derivative’s DLD cases after phecode enrichment at *P* < .05, we performed a restricted phecode enrichment analysis to test whether the associations between DLD and these phecodes could be replicated in the independent sample. For the replication analysis, the null phecode distribution for each phecode to be tested was generated from 10 000 permutations of the selected control cluster. Figure 1 depicts an overview of the analytical pipeline used in this study.

#### Sensitivity Analysis

To test the sensitivity of the phenomewide significant and independently replicated comorbidities enriched in DLD cases to the case-control matching strategy, we repeated the enrichment test under a stricter set of control-matching criteria. Specifically, we reselected controls for cases, requiring that controls must be within 3 years of the case age and the number of unique clinic visits must be within 10 visits compared with the case. We further visualized the difference in the rate of comorbidities between controls and cases by age and visit number by generating a heat map showing the log-fold change in the proportion of case-control pairs in each age or visit difference category vs the proportion of cases with the comorbidity in each age or visit difference category. A higher ratio of cases with a given comorbidity in an age group or a visit group than the proportion of data represented by that group (noted by a redder color in eTables 2 and 3 in Supplement 1) indicated that the age or visit group played a role in the significant enrichment of the comorbidity.

## Results

We conducted a comorbidity analysis using a phecode enrichment algorithm in 5273 DLD cases (1523 females [29.0%], 3748 males [71.1%], and 2 individuals of unknown sex; mean [SD] age, 16.8 [7.2] years) and 26 353 matched controls (7615 females [28.9%], and 18 729 males [71.1%], and 9 individuals of unknown sex; mean [SD] age, 14.6 [5.5] years) (Table 1). After the phecode enrichment, we filtered results to include only those phecodes that were enriched in more than 30 cases. A total of 105 phecodes were nominally associated with DLD at *P* < .05, of which 37 phecodes reached phenomewide significance at *P* < 6.2 × 10^−5^.

Phecodes associated with language and development phenotypes, auditory phenotypes, weight and nutrition phenotypes, colorectal phenotypes, conduct disorders, social disorders, motor deficits and musculature abnormalities, atopic disorders, pulmonary phenotypes, and other phenotypes (including sleep disorders and congenital anomalies of mouth or tongue) were the most associated with DLD. Details of the 37 phecodes associated with DLD are shown in Table 2, along with the simulated maximum count of the phecode observed in the controls during the 10 000 permutations. For example, the language and development phenotype included 7 phecodes: 315.2 (speech and language disorder), 264.9 (lack of normal physiological development), 315 (developmental delays and disorders), 264.3 (delayed milestones), 292.1 (aphasia or speech disturbance), 315.1 (learning disorder), and 313.2 (tics and stuttering). Similarly, auditory phenotypes included phecodes 381.11 (suppurative and unspecified otitis media) and 389.5 (disorders of acoustic nerve). Additionally, the weight and nutrition phenotypes included phecode 1002 (symptoms concerning nutrition, metabolism, and development); the pulmonary phenotypes included phecodes 512.8 (cough), 512.1 (wheezing), and 483 (acute bronchitis and bronchiolitis); colorectal phenotypes included phecode 558 (noninfectious gastroenteritis); and other phecodes such as 912 (insect bites), 473.4 (voice disturbances), and 783 (unknown fevers). eTable 1 in Supplement 1 provides complete quantile distributions of the null expected prevalence within the control set.

**Table 2.** Phecodes Associated With a DLD Case Status^a^

Phecode	Description	Count, No.	*P* value	NMax
**Language and development phenotypes**				
315.2^b^	Speech and language disorder	5046	<6.2 × 10^−5^	1085
264.9^b^	Lack of normal physiological development, unspecified	739	<6.2 × 10^−5^	324
315^b^	Developmental delays and disorders	666	<6.2 × 10^−5^	364
264.3^b^	Delayed milestones	465	<6.2 × 10^−5^	216
292.1^b^	Aphasia or speech disturbance	172	<6.2 × 10^−5^	93
315.1^b^	Learning disorder	136	<6.2 × 10^−5^	88
313.2^b^	Tics and stuttering	59	<6.2 × 10^−5^	49
**Auditory phenotypes**				
381.11	Suppurative and unspecified otitis media	726	<6.2 × 10^−5^	670
389.5^b^	Disorders of acoustic nerve	51	<6.2 × 10^−5^	45
**Weight and nutrition phenotypes**				
1002^b^	Symptoms concerning nutrition, metabolism, and development	190	<6.2 × 10^−5^	67
278.4	Abnormal weight gain	42	<6.2 × 10^−5^	20
**Colorectal phenotypes**				
558	Noninfectious gastroenteritis	213	<6.2 × 10^−5^	189
**Conduct disorders**				
313.1^b^	Attention-deficit/hyperactivity disorder	448	<6.2 × 10^−5^	302
312^b^	Conduct disorders	220	<6.2 × 10^−5^	163
**Social disorders**				
304	Adjustment reaction	147	<6.2 × 10^−5^	126
292.12	Symbolic dysfunction	30	<6.2 × 10^−5^	29
**Motor deficits and musculature abnormalities**				
350.3^b^	Lack of coordination	158	<6.2 × 10^−5^	87
781^b^	Symptoms involving nervous and musculoskeletal systems	64	<6.2 × 10^−5^	55
772	Symptoms of the muscles	62	<6.2 × 10^−5^	46
350.2	Abnormality of gait	62	<6.2 × 10^−5^	24
728.2	Laxity of ligament or hypermobility syndrome	50	<6.2 × 10^−5^	42
**Atopic disorders**				
939^b^	Atopic or contact dermatitis due to other or unspecified causes	346	<6.2 × 10^−5^	345
687.1	Rash and other nonspecific skin eruption	232	4.0 × 10^−5^	242
369.5^b^	Conjunctivitis, infectious	229	<6.2 × 10^−5^	223
112.3	Candidiasis of skin and nails	72	<6.2 × 10^−5^	72
**Pulmonary phenotypes**				
465	Acute upper respiratory infections of multiple or unspecified sites	967	<6.2 × 10^−5^	952
512.8	Cough	547	<6.2 × 10^−5^	516
483	Acute bronchitis and bronchiolitis	342	4.0 × 10^−5^	346
512.1	Wheezing	227	5.0 × 10^−5^	231
**Other phenotypes**				
783	Fevers of unknown origin	856	<6.2 × 10^−5^	749
1010	Other tests	740	<6.2 × 10^−5^	214
327^b^	Sleep disorders	227	<6.2 × 10^−5^	222
1019	Other ill-defined and unknown causes of morbidity and mortality	157	<6.2 × 10^−5^	95
750.13^b^	Congenital anomalies of mouth or tongue	135	<6.2 × 10^−5^	87
912	Insect bites	131	1.0 × 10^−5^	133
473.4	Voice disturbances	95	<6.2 × 10^−5^	60
306	Other mental disorders	92	<6.2 × 10^−5^	70

Abbreviation: DLD, developmental language disorder.

^a^ All phecodes associated with DLD cases were thus considered to be possible comorbidities of DLD. NMax was the simulated maximum count of the phecode observed in the controls during the 10 000 permutations. Count was the total number of times a phecode occurred in the cases. A phecode was significantly enriched in the cases if *P* < 6.2 × 10^−5^ (α accounting for Bonferroni correction). Information about the *International Classification of Diseases* code to phecode mapping can be accessed at https://phewascatalog.org/phecodes.

^b^ Phecodes that were also enriched in the replication analysis are listed in Table 3.

We sought replication of all 105 nominally enriched phecodes in the 3631 DLD cases and their 18 155 matched controls selected from the Geisinger EHR, an independent data set. Seventeen phecodes were replicated, and these phecodes broadly fell into the following categories: language and development phenotypes, auditory phenotypes, weight and nutrition phenotypes, conduct disorders, motor deficits and musculature abnormalities, atopic disorders (eg, dermatitis and conjunctivitis), and other phenotypes. Details of the phecodes associated with a DLD case status in the replication sample are shown in Table 3. For example, the motor deficits and musculature abnormalities category included phecodes 350.3 (lack of coordination) and 781 (symptoms involving nervous and musculoskeletal systems).

**Table 3.** Phecodes Significantly Enriched in DLD Cases in the Replication Analysis, With Results of Sensitivity Analysis^a^

Phecode	Description	Count, No.	*P* value	NMax	*P* value for sensitivity analysis
**Language and development phenotypes**					
315	Developmental delays and disorders	3631	<6.2 × 10^−5^	846	<6.2 × 10^−5^
315.2	Speech and language disorder	3631	<6.2 × 10^−5^	721	<6.2 × 10^−5^
315.1	Learning disorder	173	<6.2 × 10^−5^	106	<6.2 × 10^−5^
264.9	Lack of normal physiological development, unspecified	144	<6.2 × 10^−5^	103	<6.2 × 10^−5^
292.1	Aphasia or speech disturbance	113	<6.2 × 10^−5^	77	<6.2 × 10^−5^
264.3	Delayed milestones	94	<6.2 × 10^−5^	58	<6.2 × 10^−5^
313.2	Tics and stuttering	87	<6.2 × 10^−5^	80	<6.2 × 10^−5^
**Auditory phenotypes**					
389.5	Disorders of acoustic nerve	181	<6.2 × 10^−5^	167	<6.2 × 10^−5^
**Weight and nutrition phenotypes**					
1002	Symptoms concerning nutrition, metabolism, and development	321	<6.2 × 10^−5^	303	<6.2 × 10^−5^
**Conduct disorders**					
313.1	Attention-deficit/hyperactivity disorder	607	<6.2 × 10^−5^	403	<6.2 × 10^−5^
312	Conduct disorders	509	<6.2 × 10^−5^	340	<6.2 × 10^−5^
**Motor deficits and musculature abnormalities**					
781	Symptoms involving nervous and musculoskeletal systems	157	<6.2 × 10^−5^	89	.90
350.3	Lack of coordination	144	<6.2 × 10^−5^	75	<6.2 × 10^−5^
**Atopic disorders**					
939	Atopic or contact dermatitis due to other or unspecified cause	909	7.0 × 10^−6^	915	<6.2 × 10^−5^
369.5	Conjunctivitis, infectious	778	7.0 × 10^−6^	792	<6.2 × 10^−5^
**Other phenotypes**					
327	Sleep disorders	255	1.3 × 10^−5^	266	<6.2 × 10^−5^
750.13	Congenital anomalies of mouth or tongue	69	5.4 × 10^−5^	73	>.99

Abbreviation: DLD, developmental language disorder.

^a^ NMax was the simulated maximum count of the phecode observed in the controls during the 10 000 permutations. Count was the total number of times a phecode occurred in the cases. A phecode was significantly enriched in the cases if *P* < 6.2 × 10^−5^ (α accounting for Bonferroni correction).

The sensitivity analysis resulted in 4629 cases being matched to appropriate controls. We tested the association of the 17 independently replicated phecodes with these 4629 cases and 23 173 controls, and we repeated the phecode enrichment. Despite the lower overall sample size, we replicated the association of 15 of the 17 phecodes with DLD case status (Table 3).

## Discussion

To improve understanding of the clinical profile of children with DLD and explore the broader clinical implications of DLD, we performed discovery and replication phecode enrichment analyses to identify comorbidities of DLD in 2 independent EHRs. We found 37 phecodes that were associated with DLD, replicating previously described DLD comorbidities and identifying novel clinical associations. These phecodes included language and developmental phenotypes, auditory phenotypes, weight and nutrition phenotypes, conduct disorders, motor deficits and musculature abnormalities, and atopic disorders. Although we sought independent replication of all 105 nominally enriched phecodes, all 17 that were replicated were among the 37 phenomewide significant findings (Table 2 and Table 3).

The language and developmental phenotypes encompassed other speech and developmental disorders that were commonly observed as clinical comorbidities of DLD. All 7 phecodes (315.2, 264.9, 315, 264.3, 292.1, 315.1, and 313.2) in this category were associated with DLD in the replication analysis. Developmental language disorder is classified as a neurodevelopmental disorder, which in turn includes the umbrella of communication and language disorders. Similar to DLD, communication and language disorders are marked by delay in language learning, low vocabulary, underdeveloped sentence structures, word retrieval issues, and trouble following and understanding instructions.^36^ Children with DLD do not show impairments in the domains of nonverbal communication, pragmatics, and ability to respond to language in natural conversational settings.^37^ Given that effective use of language is important in an educational environment,^24,38,39^ children with DLD are often also diagnosed with learning disorders, such as dyslexia and dyscalculia.^19,20^

Apart from language concerns, the other disorders that were statistically prevalent in DLD cases were auditory phenotypes. Phecode 389.5 (disorders of the acoustic nerve) was associated with DLD in both the primary and the replication analyses. While there is a lack of conclusive clinical evidence, some investigations^40,41^ have found a higher prevalence of language disorders in children with a higher risk of hearing loss or with an impaired audiological profile (ie, impaired pure-tone sensitivity and issues with middle ear functions), whereas other studies^42,43,44^ have found no discernible differences in language development. Studies^45,46^ have also found underlying auditory impairments at the neural level in children with DLD, hinting that auditory processing might play a role in language development. While it is clear that hearing loss is not a factor associated with language delay in children with DLD,^11,47^ further clinical research should focus on elucidating the implications of auditory development and dysfunctions for language development.^48,49^

Other categories featured in the results were conduct disorders and social disorders. While we replicated the association between DLD and conduct disorder–related phecodes, the phecodes concerning social disorder were not replicated. In preschool-aged children who were diagnosed with language delay at age 3 years, 1 study found that 61% of the examined patients exhibited comorbidities within the domain of psychiatric and neurodevelopmental disorders, including reduced performance IQ, hyperactivity, and distractibility.^50^ Attention-deficit/hyperactivity disorder and developmental dyslexia were common comorbidities observed in children with DLD,^50,51^ although ADHD does not exacerbate language impairment.^52^ A large population-based study found that inattention played a substantial role in language delay, and language delay in turn can be associated with externalizing and aggressive behaviors,^53^ suggesting the need to examine the potential links between attention deficits and language.

We also found associations between DLD and motor deficit–related phecodes, and we replicated the association with phecodes 350.3 (lack of coordination) and 781 (symptoms involving nervous and musculoskeletal systems). Apart from the central language phenotype, motor regions (including the left frontal and basal ganglia structures,^54^ caudate nucleus, and striatum) have also been implicated in DLD.^55,56^ Neural imaging has demonstrated that the cerebellum and basal ganglia structures, which control motor learning and movement, are also recruited during the processing of language.^55,57,58,59,60,61^ This finding suggests that DLD comorbidities may not be restricted to verbal and nonverbal language development but could further extend to more generalized sensorimotor function.^62,63^ Children with DLD systematically showed weaker performance on tests of gross and fine motor skills compared with their peers,^64^ were impaired at tasks that required for them to synchronize tapping to a beat^65^; and showed delayed motor development.^66^ These observations were consistent with findings that developmental coordination disorder, which affects fine motor skills, was often comorbid with DLD.^5,67,68,69^

The other 2 sets of comorbidities that were associated with DLD were atopic disorders and sleep disorders. A meta-analysis by Xie et al^70^ revealed that children with atopic dermatitis exhibited a 62.5% higher risk of developing neurological disorders, such as ADHD, anxiety, depression, and even ASD. Strom and Silverberg^71^ reported that occurrence of eczema was associated with childhood speech disorder and that co-occurrence of eczema and ADHD or sleep disturbances had an accumulative risk for speech disorders in children. This observation was supported by Botting and Baraka,^72^ who demonstrated impaired sleep profiles both for going to sleep and waking in children with DLD. Sleep electroencephalogram studies by Dlouha et al,^68^ Picard et al,^73^ and Echenne et al^74^ have further found a prevalence of nocturnal and sleep-related epileptiform discharges in children with DLD, which can hamper neural development in childhood.^75^

The enrichment of phecodes related to congenital anomalies of the mouth and tongue might have some bearing on language development. While children with developmental anomalies of the mouth (eg, cleft palate) might face issues with articulation and speech-sound errors,^76^ some studies have shown that language proficiency (both expressive and receptive) and development of vocabulary might also be affected.^77,78,79^

The enrichment analysis identified phenotypes that have not previously been identified or described as comorbidities of DLD in the extant literature. These associated phenotypes included weight and nutrition (eg, nutrition, metabolism, and development), pulmonary (eg, cough, wheezing, and bronchitis), colorectal (eg, gastroenteritis), and other nonspecific phecodes (eg, insect bites, voice disturbances, unknown fevers).

In this study, we identified a suite of clinical comorbidities that were significantly enriched in children with DLD. The present study did not establish a causal association between DLD and the observed comorbidities, yet these results are relevant to the understanding of how DLD is clinically characterized in EHRs. The Synthetic Derivative data are generally representative of the population of Tennessee, and thus we expected the results to show minimal confounding from attrition or participation bias. As such, the novel comorbidities of DLD identified in this analysis (eg, sleep disorders, atopic disorders, and gastrointestinal issues) warrant further study. In addition, a sensitivity analysis of the 17 independently replicated DLD-associated comorbidities using stricter control-matching selection criteria validated the robustness of 15 of these comorbidities associated with the matching approach.

When the broad swath of comorbidity patterns identified in this study, which included coordination and global developmental delays, other neuropsychiatric disorders, sleep disorders, and communication impairment, is considered, it might be prudent to view DLD as a more global neurodevelopmental disorder that might have implications for several other aspects apart from language. Identifying such comorbid phenotypes may contribute to the development of a clinical risk profile, improving prevention and timely diagnostic care. This study provides a foundation for such an analysis, and future studies are needed to establish the etiology of the risk factors and comorbidities associated with DLD.

### Limitations

This study had several limitations. The statistical approach for comorbidity identification was independent of previous hypotheses and reliant only on information that can be found in EHRs, enabling the discovery of new traits associated with DLD. The associations we observed may be due to shared risk factors, may be causal, or may be spurious. Some phenotypes could not be replicated in the Geisinger sample (Table 3). These associations may be spurious or may be clinical signatures that were unique to the Vanderbilt Synthetic Derivative. Alternatively, the lack of replication could be due to the smaller size and reduced power of the Geisinger cohort.

Another potential limitation of this study was that, because there was no billing code for DLD, we deployed the APT-DLD, a previously published algorithm, to identify DLD in the medical records and separate DLD from language impairments that were precipitated by other factors. It is likely that a proportion of cases were misassigned, which may impact this analysis. To mitigate the risk of biased or spurious findings, we performed sensitivity and independent replication analyses. Another important consideration was that our approach did not assess the causal association of traits with DLD; thus, further work exploring the possible mechanisms underlying the associations we observed is needed.

## Conclusions

In this case-control study, we conducted a comorbidity analysis of DLD cases identified from EHR systems and found 37 phecodes that were associated with DLD, which replicated existing DLD comorbidities and identified novel clinical associations. By identifying and understanding these comorbidities, we may illuminate the underlying processes associated with the DLD phenotype and enhance clinical awareness of co-occurring conditions beyond language impairment in children with DLD.

## Notes

Supplement 1. **eMethods.** Application of APT-DLD to EHRs and R Script for PheCode Enrichment Algorithm **eTable 1.** Phecode Enrichment Results for All Tested Phecodes **eTable 2.** Contribution of Age Difference Between Cases and Controls to Significant Enrichment Results **eTable 3.** Contribution of Visit Difference Between Cases and Controls to Significant Enrichment Results

Supplement 2.
Data Sharing Statement

## References

[1] Tomblin JB, Records NL, Buckwalter P, Zhang X, Smith E, O’Brien M. Prevalence of specific language impairment in kindergarten children. J Speech Lang Hear Res. 1997;40(6):1245-1260. doi:10.1044/jslhr.4006.1245 9430746 PMC5075245

[2] Simms MD, Jin XM. Autism, language disorder, and social (pragmatic) communication disorder: *DSM-V* and differential diagnoses. Pediatr Rev. 2015;36(8):355-362. doi:10.1542/pir.36.8.355 26232465

[3] Norbury CF, Gooch D, Wray C, . The impact of nonverbal ability on prevalence and clinical presentation of language disorder: evidence from a population study. J Child Psychol Psychiatry. 2016;57(11):1247-1257. doi:10.1111/jcpp.12573 27184709 PMC5082564

[4] McGregor KK. How we fail children with developmental language disorder. Lang Speech Hear Serv Sch. 2020;51(4):981-992. doi:10.1044/2020_LSHSS-20-00003 32755505 PMC7842848

[5] Bishop DVM, Snowling MJ, Thompson PA, Greenhalgh T; CATALISE consortium. CATALISE: a multinational and multidisciplinary Delphi consensus study. Identifying language impairments in children. PLoS One. 2016;11(7):e0158753. doi:10.1371/journal.pone.0158753 27392128 PMC4938414

[6] Bishop DVM, Snowling MJ, Thompson PA, Greenhalgh T; and the CATALISE-2 consortium. Phase 2 of CATALISE: a multinational and multidisciplinary Delphi consensus study of problems with language development: terminology. J Child Psychol Psychiatry. 2017;58(10):1068-1080. doi:10.1111/jcpp.12721 28369935 PMC5638113

[7] DLD and Me. What is Developemental Languance Disorder? Accessed May 23, 2022. https://dldandme.org/#what-is-dld

[8] Bartak L, Rutter M, Cox A. A comparative study of infantile autism and specific development receptive language disorder—I: the children. Br J Psychiatry. 1975;126(2):127-145. doi:10.1192/bjp.126.2.127 1131465

[9] Bishop DVM. Why is it so hard to reach agreement on terminology? the case of developmental language disorder (DLD). Int J Lang Commun Disord. 2017;52(6):671-680. doi:10.1111/1460-6984.12335 28714100 PMC5697617

[10] Kamhi AG. Trying to make sense of developmental language disorders. Lang Speech Hear Serv Sch. 1998;29(1):35-44. doi:10.1044/0161-1461.2901.35 27764299

[11] Stark RE, Tallal P. Selection of children with specific language deficits. J Speech Hear Disord. 1981;46(2):114-122. doi:10.1044/jshd.4602.114 7253588

[12] Leonard LBA. 200-year history of the study of childhood language disorders of unknown origin: changes in terminology. Perspect ASHA Spec Interest Groups. 2020;5(1):6-11. doi:10.1044/2019_PERS-SIG1-2019-0007

[13] Tomas E, Vissers C. Behind the scenes of developmental language disorder: time to call neuropsychology back on stage. Front Hum Neurosci. 2019;12(January):517. doi:10.3389/fnhum.2018.00517 30687040 PMC6333853

[14] Sciberras E, Mueller KL, Efron D, . Language problems in children with ADHD: a community-based study. Pediatrics. 2014;133(5):793-800. doi:10.1542/peds.2013-3355 24753530

[15] Mueller KL, Tomblin JB. Examining the comorbidity of language disorders and ADHD. Top Lang Disord. 2012;32(3):228-246. doi:10.1097/TLD.0b013e318262010d 25505812 PMC4260529

[16] Helland WA, Helland T, Heimann M. Language profiles and mental health problems in children with specific language impairment and children with ADHD. J Atten Disord. 2014;18(3):226-235. doi:10.1177/1087054712441705 22544386

[17] Beitchman JH, Brownlie EB, Inglis A, . Seven-year follow-up of speech/language impaired and control children: psychiatric outcome. J Child Psychol Psychiatry. 1996;37(8):961-970. doi:10.1111/j.1469-7610.1996.tb01493.x 9119943

[18] Camarata SM, Gibson T. Pragmatic language deficits in attention-deficit hyperactivity disorder (ADHD). Ment Retard Dev Disabil Res Rev. 1999;5(3):207-214. doi:10.1002/(SICI)1098-2779(1999)5:3<207::AID-MRDD7>3.0.CO;2-O

[19] Pennington BF, Bishop DVM. Relations among speech, language, and reading disorders. Annu Rev Psychol. 2009;60(1):283-306. doi:10.1146/annurev.psych.60.110707.163548 18652545

[20] Manor O, Shalev RS, Joseph A, Gross-Tsur V. Arithmetic skills in kindergarten children with developmental language disorders. Eur J Paediatr Neurol. 2001;5(2):71-77. doi:10.1053/ejpn.2001.0468 11589316

[21] Morsanyi K, van Bers BMCW, McCormack T, McGourty J. The prevalence of specific learning disorder in mathematics and comorbidity with other developmental disorders in primary school-age children. Br J Psychol. 2018;109(4):917-940. doi:10.1111/bjop.12322 29974939

[22] Newbury DF, Paracchini S, Scerri TS, . Investigation of dyslexia and SLI risk variants in reading- and language-impaired subjects. Behav Genet. 2011;41(1):90-104. doi:10.1007/s10519-010-9424-3 21165691 PMC3029677

[23] Botting N. Language, literacy and cognitive skills of young adults with developmental language disorder (DLD). Int J Lang Commun Disord. 2020;55(2):255-265. doi:10.1111/1460-6984.12518 31994284

[24] Conti-Ramsden G, Durkin K, Toseeb U, Botting N, Pickles A. Education and employment outcomes of young adults with a history of developmental language disorder. Int J Lang Commun Disord. 2018;53(2):237-255. doi:10.1111/1460-6984.12338 29139196 PMC5873379

[25] Botting N, Toseeb U, Pickles A, Durkin K, Conti-Ramsden G. Depression and anxiety change from adolescence to adulthood in individuals with and without language impairment. PLoS One. 2016;11(7):e0156678. doi:10.1371/journal.pone.0156678 27404489 PMC4942141

[26] Clegg J, Hollis C, Mawhood L, Rutter M. Developmental language disorders—a follow-up in later adult life: cognitive, language and psychosocial outcomes. J Child Psychol Psychiatry. 2005;46(2):128-149. doi:10.1111/j.1469-7610.2004.00342.x 15679523

[27] Elbro C, Dalby M, Maarbjerg S. Language-learning impairments: a 30-year follow-up of language-impaired children with and without psychiatric, neurological and cognitive difficulties. Int J Lang Commun Disord. 2011;46(4):437-448. doi:10.1111/j.1460-6984.2011.00004.x 21771219

[28] Snowling MJ, Bishop DVM, Stothard SE, Chipchase B, Kaplan C. Psychosocial outcomes at 15 years of children with a preschool history of speech-language impairment. J Child Psychol Psychiatry. 2006;47(8):759-765. doi:10.1111/j.1469-7610.2006.01631.x 16898989

[29] Whitehouse AJO, Watt HJ, Line EA, Bishop DVM. Adult psychosocial outcomes of children with specific language impairment, pragmatic language impairment and autism. Int J Lang Commun Disord. 2009;44(4):511-528. doi:10.1080/13682820802708098 19340628 PMC2835860

[30] Walters CE Jr, Nitin R, Margulis K, . Automated Phenotyping Tool for Identifying Developmental Language Disorder Cases in Health Systems Data (APT-DLD): a new research algorithm for deployment in large-scale electronic health record systems. J Speech Lang Hear Res. 2020;63(9):3019-3035. doi:10.1044/2020_JSLHR-19-00397 32791019 PMC7890229

[31] American Speech Language Hearing Association. 2021 ICD-10-CM diagnosis codes related to speech, language, and swallowing disorders. Accessed January 16, 2022. https://www.asha.org/practice/reimbursement/coding/icd-10/

[32] Denny JC, Ritchie MD, Basford MA, . PheWAS: demonstrating the feasibility of a phenome-wide scan to discover gene-disease associations. Bioinformatics. 2010;26(9):1205-1210. doi:10.1093/bioinformatics/btq126 20335276 PMC2859132

[33] Failla MD, Schwartz KL, Chaganti S, Cutting LE, Landman BA, Cascio CJ. Using phecode analysis to characterize co-occurring medical conditions in autism spectrum disorder. Autism. 2021;25(3):800-811. doi:10.1177/1362361320934561 32662293 PMC7854773

[34] Pruett DG, Shaw DM, Chen H-H, . Identifying developmental stuttering and associated comorbidities in electronic health records and creating a phenome risk classifier. J Fluency Disord. 2021;68:105847. doi:10.1016/j.jfludis.2021.105847 33894541 PMC8188400

[35] Zhang T, Goodman M, Zhu F, . Phenome-wide examination of comorbidity burden and multiple sclerosis disease severity. Neurol Neuroimmunol Neuroinflamm. 2020;7(6):e864. doi:10.1212/NXI.0000000000000864 32817202 PMC7673286

[36] Sun L, Wallach GP. Language disorders are learning disabilities: challenges on the divergent and diverse paths to language learning disability. Top Lang Disord. 2014;34(1):25-38. doi:10.1097/TLD.0000000000000005

[37] Paul R, Chawarska K, Volkmar F. Differentiating ASD from DLD in toddlers. Perspect Lang Learn Educ. 2008;15(3):101-111. doi:10.1044/lle15.3.101 20852731 PMC2940236

[38] Conti-Ramsden G, Botting N, Knox E, Simkin Z. Different school placements following language unit attendance: which factors affect language outcome? Int J Lang Commun Disord. 2002;37(2):185-195. doi:10.1080/13682820110116866 12012615

[39] Laasonen M, Smolander S, Lahti-Nuuttila P, . Understanding developmental language disorder—the Helsinki longitudinal SLI study (HelSLI): a study protocol. BMC Psychol. 2018;6(1):24 "https://pubmed.ncbi.nlm.nih.gov/29301561". doi:10.1186/s40359-018-0222-7 29784061 PMC5963016

[40] Pereira MB, Befi-Lopes DM, Samelli AG. Association between audiological profile and primary language impairment in children. Int J Pediatr Otorhinolaryngol. 2015;79(1):53-57. doi:10.1016/j.ijporl.2014.11.003 25433374

[41] Samelli AG, Rondon-Melo S, Rabelo CM, Molini-Avejonas DR. Association between language and hearing disorders—risk identification. Clinics (Sao Paulo). 2017;72(4):213-217. doi:10.6061/clinics/2017(04)04 28492720 PMC5401618

[42] Klausen O, Møller P, Holmefjord A, Reisaeter S, Asbjørnsen A. Lasting effects of otitis media with effusion on language skills and listening performance. Acta Otolaryngol Suppl. 2000;543(543):73-76. doi:10.1080/000164800454026 10908983

[43] Harsten G, Prellner K, Heldrup J, Kalm O, Kornfält R. Recurrent acute otitis media: a prospective study of children during the first three years of life. Acta Otolaryngol. 1989;107(1-2):111-119. doi:10.3109/00016488909127487 2929308

[44] Harsten G, Nettelbladt U, Schalén L, Kalm O, Prellner K. Language development in children with recurrent acute otitis media during the first three years of life: follow-up study from birth to seven years of age. J Laryngol Otol. 1993;107(5):407-412. doi:10.1017/S0022215100123291 8326219

[45] Kwok EYL, Joanisse MF, Archibald LMD, Cardy JO. Immature auditory evoked potentials in children with moderate–severe developmental language disorder. J Speech Lang Hear Res. 2018;61(7):1718-1730. doi:10.1044/2018_JSLHR-L-17-0420 29974119

[46] Leppänen PHT, Lyytinen H. Auditory event-related potentials in the study of developmental language-related disorders. Audiol Neurootol. 1997;2(5):308-340. doi:10.1159/000259254 9390838

[47] Bishop DVM, Carlyon RP, Deeks JM, Bishop SJ. Auditory temporal processing impairment: neither necessary nor sufficient for causing language impairment in children. J Speech Lang Hear Res. 1999;42(6):1295-1310. doi:10.1044/jslhr.4206.1295 10599613

[48] Halliday LF, Tuomainen O, Rosen S. Auditory processing deficits are sometimes necessary and sometimes sufficient for language difficulties in children: evidence from mild to moderate sensorineural hearing loss. Cognition. 2017;166:139-151. doi:10.1016/j.cognition.2017.04.014 28577444

[49] Halliday LF, Tuomainen O, Rosen S. Language development and impairment in children with mild to moderate sensorineural hearing loss. J Speech Lang Hear Res. 2017;60(6):1551-1567. doi:10.1044/2016_JSLHR-L-16-0297 28547010

[50] Westerlund M, Bergkvist L, Lagerberg D, Sundelin C. Comorbidity in children with severe developmental language disability. Acta Paediatr. 2002;91(5):529-534. doi:10.1111/j.1651-2227.2002.tb03272.x 12113321

[51] Snowling MJ, Hayiou-Thomas ME, Nash HM, Hulme C. Dyslexia and developmental language disorder: comorbid disorders with distinct effects on reading comprehension. J Child Psychol Psychiatry. 2020;61(6):672-680. doi:10.1111/jcpp.13140 31631348 PMC7317952

[52] Redmond SM. Language impairment in the attention-deficit/hyperactivity disorder context. J Speech Lang Hear Res. 2016;59(1):133-142. doi:10.1044/2015_JSLHR-L-15-0038 26502026 PMC4867926

[53] Wang MV, Aarø LE, Ystrom E. Language delay and externalizing problems in preschool age: a prospective cohort study. J Abnorm Child Psychol. 2018;46(5):923-933. doi:10.1007/s10802-017-0391-5 29322277

[54] Ullman MT. The neural basis of lexicon and grammar in first and second language: the declarative/procedural model. Biling Lang Cogn. 2001;4(2):105-122. doi:10.1017/S1366728901000220

[55] Booth JR, Wood L, Lu D, Houk JC, Bitan T. The role of the basal ganglia and cerebellum in language processing. Brain Res. 2007;1133(1):136-144. doi:10.1016/j.brainres.2006.11.074 17189619 PMC2424405

[56] Krishnan S, Watkins KE, Bishop DVM. Neurobiological basis of language learning difficulties. Trends Cogn Sci. 2016;20(9):701-714. doi:10.1016/j.tics.2016.06.012 27422443 PMC4993149

[57] Kotz SA, Frisch S, von Cramon DY, Friederici AD. Syntactic language processing: ERP lesion data on the role of the basal ganglia. J Int Neuropsychol Soc. 2003;9(7):1053-1060. doi:10.1017/S1355617703970093 14738286

[58] Macoir J, Fossard M, Mérette C, Langlois M, Chantal S, Auclair-Ouellet N. The role of basal ganglia in language production: evidence from Parkinson’s disease. J Parkinsons Dis. 2013;3(3):393-397. doi:10.3233/JPD-130182 23948988

[59] Silveri MC. Contribution of the cerebellum and the basal ganglia to language production: speech, word fluency, and sentence construction-evidence from pathology. Cerebellum. 2021;20(2):282-294. doi:10.1007/s12311-020-01207-6 33120434 PMC8004516

[60] Groenewegen HJ. The basal ganglia and motor control. Neural Plast. 2003;10(1-2):107-120. doi:10.1155/NP.2003.107 14640312 PMC2565420

[61] Turner RS, Desmurget M. Basal ganglia contributions to motor control: a vigorous tutor. Curr Opin Neurobiol. 2010;20(6):704-716. doi:10.1016/j.conb.2010.08.022 20850966 PMC3025075

[62] Ullman MT, Pierpont EI. Specific language impairment is not specific to language: the procedural deficit hypothesis. Cortex. 2005;41(3):399-433. doi:10.1016/S0010-9452(08)70276-4 15871604

[63] Krishnan S, Asaridou SS, Cler GJ, . Functional organisation for verb generation in children with developmental language disorder. Neuroimage. 2021;226:117599. doi:10.1016/j.neuroimage.2020.117599 33285329 PMC7836232

[64] Zelaznik HN, Goffman L. Generalized motor abilities and timing behavior in children with specific language impairment. J Speech Lang Hear Res. 2010;53(2):383-393. doi:10.1044/1092-4388(2009/08-0204) 20360463 PMC3657549

[65] Corriveau KH, Goswami U. Rhythmic motor entrainment in children with speech and language impairments: tapping to the beat. Cortex. 2009;45(1):119-130. doi:10.1016/j.cortex.2007.09.008 19046744

[66] Diepeveen FB, van Dommelen P, Oudesluys-Murphy AM, Verkerk PH. Children with specific language impairment are more likely to reach motor milestones late. Child Care Health Dev. 2018;44(6):857-862. doi:10.1111/cch.12614 30155913

[67] Flapper BCT, Schoemaker MM. Developmental coordination disorder in children with specific language impairment: co-morbidity and impact on quality of life. Res Dev Disabil. 2013;34(2):756-763. doi:10.1016/j.ridd.2012.10.014 23220052

[68] Dlouha O, Prihodova I, Skibova J, Nevsimalova S. Developmental language disorder: wake and sleep epileptiform discharges and co-morbid neurodevelopmental disorders. Brain Sci. 2020;10(12):1-11. doi:10.3390/brainsci10120910 33256068 PMC7760604

[69] McGregor KK, Goffman L, Owen Van Horne A, Hogan TP, Finestack L. Developmental language disorder: applications for advocacy, research, and clinical service. Perspect ASHA Spec Interest Groups. 2020;5(1):38-46. doi:10.1044/2019_PERSP-19-00083

[70] Xie Q-W, Dai X, Tang X, Chan CHY, Chan CLW. Risk of mental disorders in children and adolescents with atopic dermatitis: a systematic review and meta analysis. Front Psychol. 2019;10:1773. doi:10.3389/fpsyg.2019.01773 31447731 PMC6691144

[71] Strom MA, Silverberg JI. Eczema is associated with childhood speech disorder: a retrospective analysis from the National Survey of Children’s Health and the National Health Interview Survey. J Pediatr. 2016;168(3):185-192.e4. doi:10.1016/j.jpeds.2015.09.066 26520915 PMC5216176

[72] Botting N, Baraka N. Sleep behaviour relates to language skills in children with and without communication disorders. Int J Dev Disabil. 2018;64(4-5):225-230. doi:10.1080/20473869.2017.1283766 34141311 PMC8115502

[73] Picard A, Cheliout Heraut F, Bouskraoui M, Lemoine M, Lacert P, Delattre J. Sleep EEG and developmental dysphasia. Dev Med Child Neurol. 1998;40(9):595-599. doi:10.1111/j.1469-8749.1998.tb15424.x 9766736

[74] Echenne B, Cheminal R, Rivier F, Negre C, Touchon J, Billiard M. Epileptic electroencephalographic abnormalities and developmental dysphasias: a study of 32 patients. Brain Dev. 1992;14(4):216-225. doi:10.1016/S0387-7604(12)80233-6 1443399

[75] Holmes GL. Effect of seizures on the developing brain and cognition. Semin Pediatr Neurol. 2016;23(2):120-126. doi:10.1016/j.spen.2016.05.001 27544468 PMC5410363

[76] Nagarajan R, Savitha VH, Subramaniyan B. Communication disorders in individuals with cleft lip and palate: an overview. Indian J Plast Surg. 2009;42(suppl):S137-S143. doi:10.4103/0970-0358.57199 19884669 PMC2825064

[77] Pamplona MC, Ysunza PA. Language proficiency in children with cleft palate. Int Arch Commun Disord. 2018;1(1):1-7. doi:10.23937/iacod-2017/1710003

[78] Broen PA, Devers MC, Doyle SS, Prouty JM, Moller KT. Acquisition of linguistic and cognitive skills by children with cleft palate. J Speech Lang Hear Res. 1998;41(3):676-687. doi:10.1044/jslhr.4103.676 9638931

[79] Lancaster HS, Lien KM, Chow JC, Frey JR, Scherer NJ, Kaiser AP. Early speech and language development in children with nonsyndromic cleft lip and/or palate: a meta-analysis. J Speech Lang Hear Res. 2019;63(1):14-31. doi:10.1044/2019_JSLHR-19-00162 31841365 PMC7213476

