# Supplementary material for: Association of Developmental Language Disorder With Comorbid Developmental Conditions Using Algorithmic Phenotyping
Source: JAMA Netw Open. 2022 Dec 29;5(12):e2248060. doi: 10.1001/jamanetworkopen.2022.48060 (PMC9857086; doi:10.1001/jamanetworkopen.2022.48060)
Supplement: Supplement 1. — eMethods. Application of APT-DLD to EHRs and R Script for PheCode Enrichment Algorithm eTable 1. Phecode Enrichment Results for All Tested Phecodes eTable 2. Contribution of Age Difference Between Cases and Controls to Significant Enrichment Results eTable 3. Contribution of Visit Difference Between Cases and Controls to Significant Enrichment Results [file jamanetwopen-e2248060-s001.pdf]

Supplemental Online Content

Nitin R, Shaw DM, Rocha DB, et al. Association of developmental language disorder with comorbid developmental conditions using algorithmic phenotyping. *JAMA Netw Open*. 2022;5(12):e2248060. doi:10.1001/jamanetworkopen.2022.48060

eMethods. Application of APT-DLD to EHRs and R Script for PheCode Enrichment Algorithm

eTable 1. Phecode Enrichment Results for All Tested Phecodes

eTable 2. Contribution of Age Difference Between Cases and Controls to Significant Enrichment Results

eTable 3. Contribution of Visit Difference Between Cases and Controls to Significant Enrichment Results

This supplemental material has been provided by the authors to give readers additional information about their work.

eMethods. Application of APT-DLD to EHRs and R Script for PheCode Enrichment Algorithm

APT-DLD is an EHR data mining algorithm which relies on the ICD codes present in EHRs and the dates of these ICD codes to classify the DLD status for a given health record. Using APT-DLD is a two-step process, the first is a Broad Search that identifies pediatric records with symptoms of language disorder by using a set of inclusion codes (presence of six language disorder ICD codes viz., 315.39, 315.32, 315.31, F80.9, F80.2, and F80.1) and a set of exclusion criteria (codes for hearing loss, intellectual disabilities, and chromosomal abnormalities). These language disorder ICD codes were selected as inclusion criteria because they are clinically used by speech language pathologists to diagnose DLD.[1] Next APT-DLD uses the additional ICD codes in the records and the dates associated with these records to sort EHRs into two categories; i) records that have language disorder symptoms accompanied by ICD codes that are known to precipitate language symptoms (e.g., presence of traumatic brain injury, epilepsy, cerebral palsy etc.) and ii) records that show presence of language disorder and <u>are not accompanied</u> by other indicators that might precipitate language issues (DLD cases). When applied to an EHR database, this algorithm allows for identification of patients with DLD with a positive predictive value (PPV) of 90-95%, as previously validated against gold-standard clinician manual coding of the EHR DLD records.[1]

1. Walters CE, Nitin R, Margulis K, et al. Automated Phenotyping Tool for Identifying Developmental Language Disorder Cases in Health Systems Data (APT-DLD): A New Research Algorithm for Deployment in Large-Scale Electronic Health Record Systems. *J Speech, Lang Hear Res*. 2020;63(9):3019-3035. doi:10.1044/2020_JSLHR-19-00397

R Script for PheCode Enrichment Algorithm

Details on the R script for phecode enrichment are provided at https://github.com/belowlab/PheCodeEnrich.

© 2022 Nitin R et al. *JAMA Network Open.*

eTable1. Phecode Enrichment Results for All Tested Phecodes

phecode	description	count.1000	p.value	p01	p05	p10	p50	p90	p95	p99	Nmax
315.2	Speech and language disorder	5046	0	907	925	935	970	1006	1016	1035	1085
465	Acute upper respiratory infections of multiple or unspecified sites	967	0	796	810	818	846	875	883	898	952
783	Fever of unknown origin	856	0	612	625	633	658	684	691	705	749
1010	Other tests	740	0	138	145	149	164	179	183	191	214
264.9	Lack of normal physiological development; unspecified	739	0	221	230	235	253	271	277	286	324
381.11	Suppurative and unspecified otitis media	726	0	537	550	557	581	606	613	627	670
315	Developmental delays and disorders	666	0	267	276	282	301	321	326	337	364
512.8	Cough	547	0	401	412	419	441	463	470	482	516
264.3	Delayed milestones	465	0	138	146	150	164	179	183	191	216
313.1	Attention deficit hyperactivity disorder	448	0	209	218	223	240	257	262	272	302
939	Atopic/contact dermatitis due to other or unspecified	346	0	249	259	264	282	300	305	315	345
369.5	Conjunctivitis; infectious	229	0	143	150	154	169	183	187	195	223
327	Sleep disorders	227	0	143	150	154	169	184	189	197	222
312	Conduct disorders	220	0	98	104	107	119	132	135	142	163
558	Noninfectious gastroenteritis	213	0	121	128	131	145	158	162	170	189
1002	Symptoms concerning nutrition; metabolism; and development	190	0	27	31	33	40	47	50	54	67
292.1	Aphasia/speech disturbance	172	0	46	50	53	62	71	74	79	93
350.3	Lack of coordination	158	0	43	47	49	58	67	69	74	87
1019	Other ill-defined and unknown causes of morbidity and mortality	157	0	46	50	53	62	71	74	79	95

© 2022 Nitin R et al. *JAMA Network Open.*

304	Adjustment reaction	147	0	70	75	78	89	100	103	109	126
315.1	Learning disorder	136	0	40	44	47	55	64	67	71	88
750.13	Congenital anomalies of mouth/tongue	135	0	37	41	43	51	59	62	67	87
473.4	Voice disturbance	95	0	23	27	29	35	42	44	48	60
306	Other mental disorder	92	0	29	33	35	42	50	52	56	70
112.3	Candidiasis of skin and nails	72	0	29	33	35	42	49	52	56	72
781	Symptoms involving nervous and musculoskeletal systems	64	0	19	22	24	30	37	39	43	55
350.2	Abnormality of gait	62	0	4	6	7	10	14	15	18	24
772	Symptoms of the muscles	62	0	15	18	19	25	31	32	36	46
313.2	Tics and stuttering	59	0	17	20	21	27	33	35	39	49
389.5	Disorders of acoustic nerve	51	0	15	18	19	25	31	32	36	45
728.2	Laxity of ligament or hypermobility syndrome	50	0	13	16	17	22	28	30	33	42
278.4	Abnormal weight gain	42	0	2	3	4	7	10	11	13	20
292.12	Symbolic dysfunction	30	0	6	8	9	13	17	18	21	29
912	Insect bite	131	1.00E-05	72	78	81	91	102	106	111	133
483	Acute bronchitis and bronchiolitis	342	4.00E-05	250	259	264	282	301	306	315	346
687.1	Rash and other nonspecific skin eruption	232	4.00E-05	158	165	169	184	199	203	212	242
512.1	Wheezing	227	5.00E-05	154	162	166	181	196	200	208	231

Note: Enrichment analysis results for all phecodes which appeared within the DLD case set. Control phecode frequency was estimated for 10,000 permutations to create a null distribution. "NMax" indicates the maximum observed frequency within the case set. "p01", "p05", "p10", "p50", "p90", "p95", "p99" indicate phecode frequency at the 1%, 5%, 10%, 50%, 90%, 99% percentiles of the null distribution.

© 2022 Nitin R et al. *JAMA Network Open.*

eTable2. Contribution of Age Difference Between Cases and Controls to Significant Enrichment Results

	Log Change	Phecode and Description													
Age Difference (control age - case age)	All_pairs	264.9 Lack of normal physiological development; unspecified	292.1 Aphasia/speech disturbance	264.3 Delayed milestones	313.2 Tics and stuttering	389.5 Disorders of acoustic nerve	1002 Symptoms concerning nutrition; metabolism; and development	313.1 Attention deficit hyperactivity disorder	312 Conduct disorders	781 Symptoms involving nervous and musculoskeletal systems	350.3 Lack of coordination	939 Atopic/contact dermatitis due to other or unspecified	369.5 Conjunctivitis; infectious	327 Sleep disorders	750.13 Congenital anomalies of mouth/tongue
-5	0	0.12	0.21	0.32	0.19	NA	0.11	0.02	0.07	0.42	0.08	0.10	0.27	0.10	0.19
-4	0	0.13	0.21	0.21	-0.19	0.10	0.18	0.00	-0.03	0.20	0.08	0.18	0.21	0.15	0.16
-3	0	0.06	0.14	0.15	0.15	0.06	0.23	0.04	-0.03	0.32	0.18	0.13	0.22	0.17	0.24
-2	0	0.06	0.25	0.14	-0.13	0.13	0.23	0.00	0.11	0.13	0.08	0.17	0.24	0.18	0.29
-1	0	0.11	0.15	0.17	0.02	0.00	0.32	0.06	0.14	0.16	0.10	0.18	0.14	0.13	0.18
0	0	0.11	0.09	0.19	0.21	-0.04	0.29	0.05	0.11	0.17	0.20	0.21	0.27	0.17	0.20
1	0	0.08	0.04	0.14	0.16	-0.01	0.11	0.11	0.08	0.18	0.07	0.20	0.19	0.09	0.10
2	0	0.11	0.05	0.07	0.08	0.06	0.12	0.11	0.05	0.08	0.07	0.09	0.08	0.13	0.09
3	0	0.04	0.01	0.03	0.00	0.01	-0.04	0.04	0.00	0.06	0.01	0.00	0.00	0.07	0.01
4	0	-0.10	-0.15	-0.18	-0.10	-0.05	-0.22	-0.07	-0.06	-0.21	-0.10	-0.14	-0.21	-0.16	-0.14
5	0	-0.13	-0.15	-0.24	-0.07	0.01	-0.34	-0.07	-0.06	-0.39	-0.14	-0.26	-0.34	-0.23	-0.34

Note: Log fold change of the ratio of the proportion of case-control pairs within each age difference bin with a specific phecode compared to the proportion of all case-control pair across each age difference groups. Intensity of red coloring indicates that the log fold change is further from zero, and green coloring indicates log fold change is closer to zero. Log fold changes that deviate further from zero indicates a greater contribution of age to the significant enrichment result

© 2022 Nitin R et al. *JAMA Network Open.*

eTable3. Contribution of Visit Difference Between Cases And Controls to Significant Enrichment Results

	Log Change	Phecode and Description													
Visit Count	All_pairs	264.9 Lack of normal physiological development; unspecified	292.1 Aphasia/speech disturbance	264.3 Delayed milestones	313.2 Tics and stuttering	389.5 Disorders of acoustic nerve	1002 Symptoms concerning nutrition; metabolism; and development	313.1 Attention deficit hyperactivity disorder	312 Conduct disorders	781 Symptoms involving nervous and musculoskeletal systems	350.3 Lack of coordination	939 Atopic/contact dermatitis due to other or unspecified	369.5 Conjunctivitis; infectious	327 Sleep disorders	750.13 Congenital anomalies of mouth/tongue
-16 to -20	0	0.643332057	NA	0.688596871	1.32270409	NA	1.117383212	1.267902011	1.277084055	#NUM!	1.327193853	1.056573656	1.357466195	0.290656862	0.514040223
-11 to -25	0	0.61370959	0.540609435	0.106132435	NA	NA	1.154707535	0.685437576	0.393589624	0.964845838	1.01773069	0.474109221	0.962088403	0.708192427	0.630545792
-6 to -10	0	-0.157037181	0.059206901	-0.074240103	0.06768211	-0.01962705	0.270401573	0.030404382	0.1718244	-0.046066021	0.04922268	0.310675104	0.307055209	0.19079224	0.118474438
-1 to -5	0	-0.079919151	0.000691173	-0.030863417	0.00719679	0.01203385	0.063138785	-0.017207983	0.026969721	-0.001041157	-0.04785922	0.036083372	0.060755385	0.075520112	-0.002126523
0	0	0.173037939	0.155455111	0.172545262	-0.01289146	0.08885562	-0.206313109	0.054522345	-0.030482766	0.114804716	0.095686906	-0.004094695	-0.171253948	-0.06721508	0.081534666
1 to 5	0	-0.02998598	-0.076144508	-0.075764578	-0.10641932	-0.04330165	-0.151329145	-0.099720797	-0.137500945	-0.06818957	-0.12444919	-0.209591512	-0.225241134	-0.12661728	-0.105738418
6 to 10	0	0.089797337	-0.001619155	0.083428948	0.1254604	0.03091282	0.04998559	0.120932591	0.091526121	0.072101889	0.134857495	0.053465725	0.094791576	0.038792218	0.074744394
-11 to -25	0	NA	NA	-0.080954208	NA	NA	NA	NA	NA	NA	NA	NA	NA	NA	NA
-16 to -20	0	NA	NA	-0.433136726	NA	NA	-0.083531631	NA	NA	NA	0.001340273	-0.366189936	#NUM!	NA	NA

Note: Log fold change of the ratio of the proportion of case-control pairs within each visit difference bin with a specific phecode compared to the proportion of all case-control pair across each visit difference groups. Intensity of red coloring indicates that the log fold change is further from zero, and green coloring indicates log fold change is closer to zero. Log fold changes that deviate further from zero indicates a greater contribution of clinical visit count to the significant enrichment result
