# Supplementary material for: Association of Developmental Language Disorder With Comorbid Developmental Conditions Using Algorithmic Phenotyping
Source: JAMA Netw Open. 2022 Dec 29;5(12):e2248060. doi: 10.1001/jamanetworkopen.2022.48060 (PMC9857086; doi:10.1001/jamanetworkopen.2022.48060)
Supplement: Supplement 2. — Data Sharing Statement [file jamanetwopen-e2248060-s002.pdf]

Data Sharing Statement

Nitin. Association of Developmental Language Disorder With Comorbid Developmental Conditions Using Algorithmic Phenotyping. *JAMA Netw Open*. Published December 29, 2022. doi:10.1001/jamanetworkopen.2022.48060

Data
Data available: No

Additional Information
Explanation for why data not available: We do not have permission to share individual level patient data.
